# An Account of the Practice of One of the Physicians of the Westminster General Dispensary, and of the Western Dispensary, from the 20th of February, to the 20th of March, 1808

**Published:** 1808-04

**Authors:** Samuel Fothergill

**Affiliations:** Leicester Square


*An Account of the Practice of one of the Physicians of the Westminster General Dispensary, and of the Western Dispensary, from the 20th of February, to the 20th of March,* 1808.

ACUTE DISEASES.

| | |
|---|---|
| Catarrh | 5 |
| Peripneumony | 2 |
| Pleurisy | 1 |
| Acute Rheumatism | 4 |
| Fever | 3 |
| Small-pox | 1 |
| Measles | 8 |
| Erysipelas | 1 |
| Acute Diseases of Infants | 7 |

CHRONIC DISEASES.

| | |
|---|---|
| Cough and Dyspnœa | 40 |
| Ditto with Debility after Measles | 6 |
| Pulmonary Consumption | 6 |
| Peripneumonia Notha | 4 |
| Pleurodyne | 3 |
| Asthma | 2 |
| Chronic Rheumatism | 12 |
| Lumbago and Sciatica | 5 |
| Cephalalgia | 4 |
| Hydrocephalus | 1 |
| Mania | 1 |
| Asthenia | 12 |
| Marasmus | 2 |
| Tabes Mesenterica | 1 |
| Paralysis | 4 |
| Dropsy | 5 |
| Gout | 2 |
| Dyspepsia | 6 |
| Epilepsy | 1 |
| St. Vitus's Dance | 1 |
| Gastrodynia | 8 |
| Diarrhœa | 9 |
| Bilious Vomiting | 3 |
| Gravel and Dysure | 2 |
| Scurvy | 3 |
| Cutaneous Eruption | 9 |
| Hysteria | 2 |
| Chlorosis | 3 |
| Amenorrhœa | 5 |
| Menorrhagia | 1 |
| Leucorrhœa | 2 |

I have had occasion to observe during the course of these Reports, that in my attendance upon the poor in Westminster, I have for the last two years met with very few cases of natural small-pox. The one now recorded is that of an adult, who received the infection at a village in Lincolnshire, where the complaint was very prevalent in January last; soon after his return to London he sickened, and went through the disease very well.

Besides the numerous, severe, and dangerous pulmonic affections, which have constantly demanded the closest attention during the last month, rheumatic complaints have been frequent and difficult to combat. This disease, whether in its acute or in its chronic form, is peculiarly distressing to the patient, long in its progress, and frequently baffles every attempt at alleviation or cure. The poor, industrious, and labouring part of the community, from their greater exposure to the changes of weather, their neglect of precaution, want of warm cloathing and comfortable habitations, are the most subject to this disease, ease, which in its chronic state frequently incapacitates them for months from pursuing their accustomed employments; and their families are consequently reduced to the extreme of misery.

When the different combinations of bark, guaiacum, opium, mercury, and antimony have proved ineffectual, I have sometimes found speedy good effects from the cautious exhibition of Fowler's Solution of Arsenic; and I know of no objection to its use but the name. Patients are often extremely alarmed when they find they have been taking arsenic, and are very ready to attribute any future illness which may befall them, to the use of that mineral. I have never yet seen any seriously bad consequences follow, even when it has been taken for several days successively; an over dose is apt to occasion a sense of heat about the fauces and in the stomach; and sometimes the bowels are affected with diarrhœa; but this is easily remedied. I usually give equal parts of laudanum, in any convenient menstruum, and have never given more than fifteen drops of the solution for a dose; ten will generally be as much as the patient can bear, repeated three times a-day.

This remedy is particularly noticed and recommended in a recent publication, containing much valuable practical information;* but the practice of giving it in chronic rheumatism is by no means confined, as the author intimates, to a few members of the profession residing in Manchester; I have occasionally used it during the last three years, and believe, that many other practitioners in London also give it in some cases of chronic rheumatism; but as it frequently fails, its use will never become general. From the few trials I have made with this medicine, (substituting the word *chiefly* for only) I entirely concur with the following observations of Dr. Bardsley, "that it is *only* in the protracted chronic rheumatism, where the vital powers are much diminished, and the ends of the bones, periosteum, capsules, or ligaments of the joints, are likewise partially affected, that the use of arsenic is likely to prove eminently successful."

SAMUEL FOTHERGILL.

*Leicester Square.*

---

* [illegible]r. Bardsley's Medical Reports.

